# The Impact of Comorbid Diabetes on Short-Term Postoperative Outcomes in Stage I/II Colon Cancer Patients Undergoing Open Colectomy

**DOI:** 10.1155/2020/2716395

**Published:** 2020-08-04

**Authors:** Ko-Chao Lee, Kuan-Chih Chung, Hong-Hwa Chen, Kung-Chuan Cheng, Kuen-Lin Wu, Ling-Chiao Song, Wan-Hsiang Hu

**Affiliations:** ^1^Division of Colorectal Surgery, Department of Surgery, Chang Gung Memorial Hospital-Kaohsiung Medical Center, Chang Gung University College of Medicine, No. 123, Dapi Rd. Niaosong District, Kaohsiung City 83301, Taiwan; ^2^Department of Information Management & College of Liberal Education, Shu-Te University, No. 59, Hengshan Rd., Yanchao, Kaohsiung City 82445, Taiwan; ^3^Department of Anesthesiology, Chang Gung Memorial Hospital-Kaohsiung Medical Center, Chang Gung University College of Medicine, No. 123, Dapi Rd. Niaosong District, Kaohsiung City 83301, Taiwan; ^4^Division of Colon & Rectal Surgery, Department of Surgery, E-DA Hospital, I-Shou University, No. 1, Sec. 1, Syuecheng Rd., Dashu District, Kaohsiung City 84001, Taiwan

## Abstract

**Purpose:**

This study aimed at evaluating the impact of comorbid diabetes on short-term postoperative outcomes in patients with stage I/II colon cancer after open colectomy.

**Methods:**

The data were extracted from the National Inpatient Sample database (2005-2010). Short-term surgical outcomes included in-hospital mortality, postoperative complications, and hospital length of stay.

**Results:**

A total of 49,064 stage I/II colon cancer patients undergoing open surgery were included, with a mean age of 70.35 years. Of them, 21.94% had comorbid diabetes. Multivariable analyses revealed that comorbid diabetes was significantly associated with a lower risk of in-hospital mortality and postoperative complications. Compared to patients without diabetes, patients with uncomplicated diabetes had lower percentages of in-hospital mortality and postoperative complications, but patients with complicated diabetes had a higher percentage of postoperative complications. In addition, patients with diabetes only, but not patients with diabetes and hypertension only, had a lower percentage of in-hospital mortality than patients without any comorbidity.

**Conclusion:**

The present results suggested the protective effects of uncomplicated diabetes on short-term surgical outcomes in stage I/II colon cancer patients after open colectomy. Further studies are warranted to confirm these unexpected findings and investigate the possible underlying mechanisms.

## 1. Introduction

Colon cancer is the third most commonly diagnosed cancers in the United States (US) and the fourth leading cause of cancer-related death worldwide; colon cancer is associated with a high recurrence rate and poor prognosis [1, 2]. Open colectomy is the most common surgical treatment modality for colon cancer [3]. According to a European multicenter randomized trial, patients undergoing open colectomy for stage I-III colon cancer had a mean hospital length of stay (LOS) of 9.3 ± 7.3 days and a postoperative complication rate of 20% in [4]. A Japanese randomized controlled trial found that for the periods of 2004-2005, 2006-2007, and 2008-2009, the mean lengths of hospital stay were 12, 11, and 11 days, respectively, and postoperative complication rates were 27.6%, 20.3%, and 21.3%, respective, in patients with stage II/III colon cancer after open surgery [5]. Furthermore, an analysis of American College of Surgeons National Surgical Quality Improvement Program (ACS-NSQIP) data revealed that in colon cancer patients undergoing open surgery, the postoperative complication rate was 25.3%, the mean hospital LOS was 9.5 ± 6.3 days, and 30-day mortality rate was 2.9% [6].

It is estimated that there are 463 million people with diabetes worldwide in 2019, and the global diabetes prevalence is projected to increase from 9.3% to 10.7% by 2045 [7]. Comorbidity is associated with adverse outcomes in colon cancer patients after surgery, and diabetes is one of the most common comorbidities [8, 9]. Among colon cancer patients, the diabetes prevalence rates were 10.9%, 11.4%, and 16.8% in New Zealand, England, and Australia, respectively [10–12]. However, most studies that investigated the effect of comorbid diabetes on short-term surgical outcomes in colorectal cancer did not distinguish colon cancer from rectal cancer [13–16]. Notably, a population-based study specifically focusing on stage I-III colon cancer revealed that comorbid diabetes is associated with elevated risk of postoperative complications in patients 80 and older after either open resection or laparoscopic resection in Finland [17].

According to the tumor node metastasis (TNM) staging system, stage I and II colon cancers are lymph node-negative diseases, while stage III and IV colon cancer are lymph node-positive [18]. Since lymph node metastasis affects long-term prognosis in colon cancer [18, 19], short-term surgical outcomes are also likely to be influenced by lymph node involvement. We assumed that comorbid diabetes may exert differential effects on short-term surgical outcomes in between node-negative colon cancer and node-positive colon cancer. Hence, the present population-based study was designed to evaluate associations between comorbid diabetes and short-term postoperative outcomes specifically in stage I/II colon cancer patients undergoing open colectomy. Short-term surgical outcomes, such as in-hospital mortality, postoperative complications, and hospital LOS, were systematically examined. In addition, subgroup analyses were conducted to gain a thorough overview of the influences of manifestation of diabetes and multimorbidity on short-term postoperative outcomes in stage I/II colon cancer after open surgery.

## 2. Materials and Methods

### 2.1. Data Source

The National Inpatient Sample (NIS) database (2005-2010) was utilized for this retrospective cross-sectional population-based study. The NIS database is the largest inpatient health care database in the United States (US), which is maintained by the Agency for Healthcare Research and Quality as part of the Healthcare Cost and Utilization Project (HCUP). This database covers de-identified demographic and clinical data on approximately seven million hospital stays each year nationwide, which is equivalent to a 20% stratified sample of the American community hospitals.

Due to de-identified information in the NIS database, the requirement for Institutional Review Board (IRB) approval and informed consent from each participant was waived. While, the data reported in this study conform to the Data Use Agreement for the NIS with certificate number (HCUP-5VW28M94I).

### 2.2. Study Population

The International Classification of Diseases, 9^th^ Revision (ICD-9-CM) procedural codes were used to identify all patients in the NIS between 2005 and 2010 who met the following criteria: (i) was diagnosed with colon cancer (ICD-9-CM code: 153) and with AJCC stage I/II (DS_Stage1 = 2.01, 2.02); and (ii) underwent open colectomy (ICD-9-CM code: 45.7). In addition, ICD-9-CM 250 diagnostic code was utilized to identify all patients with either type I or type II diabetes. These diabetic patients were then classified into two groups: uncomplicated diabetes (ICD-9-CM 250.0) and complicated diabetes that is diabetes with renal, ophthalmological, neurological, or other manifestations (ICD-9-CM codes: 250.1-250.9) [14].

### 2.3. Outcomes and Variables

The short-term surgical outcomes in the present study included in-hospital mortality, postoperative complications, and hospital LOS. Three categories of postoperative complications were identified by ICD-9-CM Codes: wound complications (998.3, 998.1, 879.2), medical complications (410: acute myocardial infarction, 411.81: acute coronary occlusion without myocardial infarction, 427.9: cardiac dysrhythmia, 518.0: pulmonary collapse, 518.81: acute respiratory failure), and surgical complications (560.1: paralytic ileus, 567: peritonitis and retroperitoneal infections, 568.81: nontraumatic hemoperitoneum, 998.5: postoperative infection).

The independent variables included demographic characteristics (age, sex, race/ethnicity, and household income), hospital information, severity of illness, and comorbidities. The severity of illness is defined as the extent of loss of organ function based on the patient's overall condition, but not the severity of any specific disease. The severity of illness is classified into 4 subclasses, including minor, moderate, major, and extreme loss of function. Due to unequal sample sizes among 4 subclasses, patients with minor or moderate loss of function were pooled together, and patients with major or extreme loss of function were combined in the present study. Comorbidities included hypertension, congestive heart failure, obesity, peripheral vascular disorder, pulmonary circulation disorders, renal failure, and other cancer.

### 2.4. Statistical Analysis

Descriptive statistics of the patients were presented as unweighted number (*n*) and weighted percentage (%). Differences in categorical variables between groups were examined by chi-square test. Univariate and multivariable logistic regression models were performed to identify the risk factors associated with short-term surgical outcomes. The variables that were significant in the univariate regression model were adjusted for in the multivariable logistic regression analysis, and diabetic patients were classified into two groups depending on the presence of any manifestation of diabetes. The chi-square tests were used to examine the differences in the percentages of short-term surgical outcomes between patients with and without various comorbid conditions. Weighted samples (DISCWT), stratum (NIS_STRATUM), and cluster (HOSPID) were used to produce national estimates for all analyses. A two-sided *p* value < 0.05 in the statistical process was considered significantly different. Statistical analyses were performed using the SAS V.9.4 software (SAS Institute Inc., Cary, NC, USA).

## 3. Results

### 3.1. Baseline Characteristics of Stage I/II Colon Cancer Patients Undergoing Open Colectomy

A total of 49,064 AJCC stage I/II colon cancer patients who underwent open colectomy between 2005 and 2010 were included in this study. The baseline characteristics of the included patients are summarized in Table 1. The mean age of the patients was 70.35 years old. The majority of the patients (52.88%) were female, and 60.30% were white. Most patients had minor and moderate loss of function (72.76%). In the total population, the most common comorbidity was hypertension (57.4%), followed by diabetes (21.94%), congestive heart failure (9.1%), obesity (7.7%), and renal failure (5.3%). The majority of the diabetic patients did not have any diabetes-derived manifestation (*n* = 9,781, 90.93%). Notably, diabetic patients had significantly higher prevalence rates of hypertension, chronic heart failure, obesity, peripheral vascular disease, and renal failure than nondiabetic patients.

### 3.2. Short-Term Postoperative Outcomes in Diabetic and Nondiabetic Patients

In Table 2, the short-term surgical outcomes of colon cancer patients with and without comorbid diabetes are summarized. There were no significant differences in the percentages of in-hospital mortality and total postoperative complication rate between colon cancer patients with and without comorbid diabetes. However, diabetic patients had a significantly higher percentage of medical complications, but a significantly lower percentage of surgical complications compared to nondiabetic patients. Furthermore, diabetic patients had a significantly longer hospital LOS and significantly higher percentages of hospital LOS > 7 days, >10 days, and >14 days than nondiabetic patients did.

### 3.3. Factors Associated with In-Hospital Mortality

After adjusting for diabetes and the significant variables identified in the univariate logistic regression analyses, multivariable analyses revealed that comorbid diabetes [adjusted odds ratio (aOR) = 0.70], female (aOR = 0.74), and the Q4 (76^th^ to 100^th^ percentile) household income (Q4 vs. Q1, aOR = 0.75) were significantly associated with a lower risk of in-hospital mortality in stage I/II colon cancer patients after open surgery, while older age was significantly associated with an elevated risk of in-hospital mortality (aOR = 1.05) (Table 3). Compared to minor and moderate loss of function, patients with major and extreme loss function had an increased risk of in-hospital mortality (aOR = 19.60). Regarding comorbidities, stage I/II colon cancer patients with congestive heart failure (aOR = 1.30), peripheral vascular disorders (aOR = 1.08), pulmonary circulation disorders (aOR = 1.08), or renal failure (aOR = 1.34) had significantly higher risk of in-hospital mortality, as compared to the patients without the corresponding comorbidity. However, patients with obesity had a significantly lower risk of in-hospital mortality (aOR = 0.50) (Table 3).

### 3.4. Factors Associated with Postoperative Complications

After adjusted for diabetes and the significant variables identified in the univariate analyses, multivariable logistic regression analyses revealed that comorbid diabetes (aOR = 0.87), female (aOR = 0.75), Hispanics (Hispanic vs. White, aOR = 0.88), and hospitals with smaller bed size (small vs. large, aOR = 0.91) were significantly associated with a lower risk of postoperative complications in stage I/II colon cancer patients after open colectomy (Table 4). In contrast, older age was significantly associated with an increased risk of postoperative complications (aOR = 1.00). Compared to the hospital region in the Northeast, patients from the hospitals in the other regions were significantly associated with an elevated risk of postoperative complications (Midwest, aOR = 1.25; South, aOR = 1.26; West, aOR = 1.39). Patients with major and extreme loss function had a significantly higher risk of postoperative complications than those with minor and moderate loss function (aOR = 5.60). In addition, hypertension, congestive heart failure, peripheral vascular disorders, pulmonary circulation disorders, and renal failure were significantly associated with lower risk of postoperative complications (aOR = 0.92, 0.68, 0.80, 0.76, and 0.78, respectively) (Table 4).

### 3.5. Factors Associated with Hospital LOS More than 7 Days

After adjustment for diabetes and the significant variables that were identified in the univariate analyses, multivariable analyses revealed that comorbid diabetes was not significantly associated with the risk of hospital LOS > 7 days in stage I/II colon cancer patients undergoing open colectomy (Table 5). However, female (aOR = 0.94) and the higher household income (Q2 vs. Q1, aOR = 0.86; Q3 vs. Q1, aOR = 0.81; Q4 vs. Q1, aOR = 0.74) were significantly associated with lower odds of hospital LOS > 7 days. In contrast, older age (aOR = 1.03) and race (Black vs. White, aOR = 1.48; Hispanic vs. White, aOR = 1.40; others vs. White, aOR = 1.26) were significantly associated with higher odds of hospital LOS > 7 days. The hospitals with small bed size had a significantly lower odds of hospital LOS > 7 days (small vs. large, aOR = 0.87). Compared to the hospital region in the Northeast, the other regions were significantly associated with lower odds of hospital LOS > 7 days (Midwest, aOR = 0.89; South, aOR = 0.93; West, aOR = 0.74). Patients with major and extreme loss functions were significantly associated with a higher risk of hospital LOS > 7 days compared to those with minor and moderate loss function (aOR = 8.32). Moreover, hypertension, congestive heart failure, and renal failure were significantly associated with a lower risk of hospital LOS > 7 days (aOR = 0.90, 0.95, and 0.88, respectively); however, other cancer was significantly associated with a higher risk of hospital LOS > 7 days (Table 5).

### 3.6. Impact of Diabetes on Short-Term Postoperative Outcomes Stratified by Severity of Illness

The short-term postoperative outcomes in stage I/II colon cancer patients with or without diabetes stratified by severity of illness are summarized in Table 6. Among stage I/II colon cancer patients with minor and moderate loss of function, patients with diabetes had significantly lower percentages of postoperative complications and hospital LOS > 7 days than patients without diabetes. On the other hand, among stage I/II colon cancer patients with major and extreme loss function, patients with diabetes had significantly lower percentages of in-hospital mortality, postoperative complications, and hospital LOS > 7 days than those without diabetes (Table 6).

### 3.7. Impact of Manifestation of Diabetes on Short-Term Surgical Outcomes

The short-term postoperative outcomes in stage I/II colon cancer patients without diabetes, with uncomplicated diabetes, and with complicated diabetes are summarized in Table 7. Patients with uncomplicated diabetes had a significantly lower percentage of in-hospital mortality than patients without diabetes. However, there was no significant difference in the percentage of in-hospital mortality in between patients without diabetes and patients with complicated diabetes. Compared to patients without diabetes, patients with uncomplicated diabetes had a significantly lower percentage of postoperative complications, while patients with complicated diabetes had a significantly higher percentage of postoperative complications. Furthermore, patients with uncomplicated or complicated diabetes had significantly higher percentages of hospital LOS > 7 days than patients without diabetes (Table 7).

### 3.8. Impact of Multimorbidity on Short-Term Surgical Outcomes

The short-term postoperative outcomes in stage I/II colon cancer patients without any comorbidity, with diabetes only, and with diabetes and hypertension only (multimorbidity) are summarized in Table 8. Patients with diabetes only had a significantly lower percentage of in-hospital mortality than patients without any comorbidity. The percentages of hospital LOS > 7 days were significantly higher in both patients with diabetes only and patients with diabetes and hypertension only, compared to that of patients without comorbidity. In addition, the percentage of hospital LOS > 7 days was higher in patients with diabetes and hypertension than in patients with diabetes only. Nevertheless, there were no differences in the percentage of postoperative complications between groups (Table 8).

## 4. Discussion

Although Niemelainen et al. [17] previously reported that comorbid diabetes increases the likelihood of postoperative complications in older patients with stage I-III colon cancer undergoing open or laparoscopic surgery [17], the present analysis of the NIS (2005-2010) unexpectedly revealed that comorbid diabetes was significantly associated with lower risks of not only postoperative complications but also in-hospital mortality in stage I/II colon cancer patients undergoing open colectomy. Patients with uncomplicated diabetes had significantly lower percentages of in-hospital mortality and postoperative complications than those without diabetes, but patients with complicated diabetes had a significantly higher percentage of postoperative complications than those without diabetes, suggesting that the putative protective role of comorbid diabetes may be mainly contributed by uncomplicated diabetes. Moreover, patients with diabetes only (single comorbidity), but not patients with diabetes and hypertension only (multimorbidity), had a significantly lower percentage of in-hospital mortality than patients without any comorbidity.

On the other hand, inconsistent results regarding the effect of comorbid diabetes on hospital LOS > 7 days were found in the present study. Multivariable analysis revealed no association between comorbid diabetes and hospital LOS > 7 days in stage I/II colon cancer patients after open colectomy. Nevertheless, the percentages of hospital LOS > 7 days were 37.97%, 41.10%, and 53.65% in colon cancer patients without diabetes, with uncomplicated diabetes, and with complicated diabetes, respectively. Patients with diabetes and hypertension only had the highest percentage of hospital *LOS* > 7 days (43.31%), followed by those with diabetes only (37.31%) and without any comorbidity (32.62%). Hence, comorbid diabetes likely increases the probability of hospital LOS > 7 days, and the presence of manifestation of diabetes or hypertension may further raise the possibility of hospital LOS > 7 days.

The present study identified that 21.94% of stage I/II colon cancer patients undergoing open colectomy had comorbid diabetes in the US. The diabetes prevalence rate in our study population seems to be higher than those previously reported in New Zealand, England, and Australia (10.9% to 16.8%) [10–12]; however, these previous studies did not specifically focus on stage I/II colon cancer and open colectomy, so no fair comparison can be made. Furthermore, Sarfati et al. [10] reported that among colon cancer patients with comorbid diabetes, 54.13% of them had uncomplicated diabetes and 45.87% had complicated diabetes, although no information about cancer stage was available in their study. In contrast, 90.93% of stage I/II colon cancer patients with comorbid diabetes had uncomplicated diabetes in the present study.

Notably, multiple lines of indirect evidence did not support the putative protective role of comorbid diabetes in postoperative complications and in-hospital mortality observed in the present study. Obese patients with stage I/II colon cancer are more likely to have comorbid diabetes and develop postoperative complications [20]. Colorectal cancer patients with comorbid diabetes have an elevated risk of 30-day mortality following colorectal surgery [16]. Comorbid diabetes is a risk factor for developing surgical complications in stage I-IV colorectal cancer patients after open or laparoscopic surgery, but has no effect on medical complications and 30-day mortality [12]. A study of the NIS (2006-2008) found no association between comorbid diabetes and in-hospital mortality in colorectal cancer patients undergoing open or laparoscopic surgery [15]. Furthermore, in colorectal cancer patients after laparoscopic surgery, comorbid diabetes is associated with 30-day overall morbidity, cardiac and septic complications, and longer hospital LOS [21].

On the other hand, Anand et al. analyzed the NIS (1998-2005) and unanticipatedly found that comorbid diabetes was significantly associated with a lower risk for in-hospital mortality in colorectal cancer patients undergoing open or laparoscopic surgery [14]. After stratification, uncomplicated diabetes was still a protective factor for in-hospital mortality regardless of the type of hospital admission; however, complicated diabetes was turned out to be a risk factor for elective admission, but not for emergency admission [14]. In addition, all diabetes and uncomplicated diabetes were significantly associated with a reduced risk of postoperative complications [14]. Hence, uncomplicated diabetes may exert protective effects on in-hospital mortality and postoperative complications in colorectal cancer patients [14], which are similar to our unforeseen findings in stage I/II colon cancer patients after open colectomy.

Anand et al. [14] proposed a possible explanation for their paradoxical findings. The prevalence of hyperglycemia in hospitalized patients may be dramatically underestimated [22], and hyperglycemia is associated with an elevated risk for in-hospital mortality and longer hospital LOS [23]. Therefore, Anand et al. [14] hypothesizes that some colorectal cancer patients without comorbid diabetes identified in the NIS may have undiagnosed hyperglycemia. Cancer patients without diabetes but with undiagnosed hyperglycemia may confound the statistical results of the studies using retrospective administrative database, including the present study of the NIS (2005-2010).

Furthermore, comorbidities other than diabetes may exert confounding effects on short-term postoperative outcomes. Besides diabetes, hypertension, chronic obstructive pulmonary disease (COPD), and congestive heart failure were the most common comorbidities in colon cancer patients worldwide [9–11, 24]. Notably, the prevalence of multimorbidity increased with time in colorectal cancer patients [24], and multimorbidity doubled the risk of six-month mortality in stage I-IV colorectal cancer patients [25]. In the present study, 59.27% of stage I/II colon cancer patients without comorbid diabetes had one or more other comorbid conditions; 86.39% of colon cancer patients with comorbid diabetes had one or more additional comorbidities. After using colon cancer patients without any comorbidity as the reference group in the present study, the results of percentage analyses (Table 7) appear to be more clinically reasonable, except for the lower percentage of in-hospital mortality in patients with diabetes only compared to that of patients without any comorbidity.

In addition, the use of antidiabetic medications may be a potential confounder. Metformin is a widely used antidiabetic drug for management of type 2 diabetes mellitus, and survival benefit of metformin adjuvant treatment has been suggested in patients with colorectal cancer or prostate cancer [26], pancreatic cancer [27], or thyroid cancer [28], which may be attributed to the anticancer effects of metformin [29]. The putative protective effects of uncomplicated diabetes on short-term surgical outcomes in stage I/II colon cancer patients observed in the present study may be in part due to the long-term use of metformin by colon cancer patients with uncomplicated diabetes. However, against the above hypothesis, Bae et al. found that diabetes medication did not affect cancer recurrence or survival in stage II colon cancer patients [30].

The present cross-sectional population-based study has certain limitations. The present study analyzed the NIS database from 2005 to 2010, because the NIS dropped cancer staging (DS_DX_Category1) in 2011. The NIS database provides only in-hospital outcomes, so the present study could not address long-term mortality and morbidity. No medication information was available in the NIS; hence, the hypothesis about antidiabetic drugs could not be tested. The present study used the ICD-9 codes to characterize the disease and postoperative complications, while the errors and failure in ICD-9 coding have been reported in which potential inaccuracies may occur [31]. The severity of comorbidities was unknown based on the ICD-coded system in which might confound the results. The cross-sectional design of this study can only demonstrate associations; therefore, causation cannot be determined. Finally, the NIS database collected inpatient information in the US, thus; therefore, additional studies with larger sample sizes conducted in other geographic regions are warranted to confirm the current findings.

## 5. Conclusion

The present study unexpectedly suggested that comorbid uncomplicated diabetes reduces in-hospital mortality and postoperative complications in stage I/II colon cancer patients undergoing open colectomy. The current findings also suggested that colon cancer patients without any comorbidity and comorbidity other than diabetes should be taken into consideration while designing the relevant research. Further large-scale cohort studies with more restricted study designs are essential to confirm the current paradoxical findings and investigate the possible underlying mechanisms.

## Figures and Tables

**Table 1:** Baseline characteristics of the stage I/II colon cancer patients with or without diabetes.

Variables, *n* (weighted %)	Total	Diabetes		*p* value
		No	Yes	
	*n* = 49,064	*n* = 38,301	*n* = 10,763	
	(Weighted *N* = 242,136)	(Weighted *N* = 189,017)	(Weighted *N* = 53,119)	
Demographic features				
Age, year, mean ± SE	70.35 ± 0.08	70.02 ± 0.09	71.56 ± 0.10	<0.0001^∗^
Female	25937 (52.88)	20556 (53.69)	5381 (50.0)	<0.0001^∗^
Race				<0.0001^∗^
White	29532 (60.30)	23430 (61.27)	6102 (56.84)	
Black	3716 (7.54)	2612 (6.77)	1104 (10.27)	
Hispanic	2339 (4.69)	1662 (4.28)	677 (6.15)	
Other ethnic groups^a^	2089 (4.25)	1587 (4.14)	502 (4.64)	
Unknown	11388 (23.22)	9010 (23.54)	2378 (22.10)	

Household income				<0.0001^∗^
Q1	12153 (24.69)	9147 (23.79)	3006 (27.89)	
Q2	12955 (26.38)	9947 (25.95)	3008 (27.92)	
Q3	11933 (24.36)	9461 (24.74)	2472 (23.02)	
Q4	11045 (22.58)	8965 (23.49)	2080 (19.36)	
Unknown	978 (1.98)	781 (2.03)	197 (1.81)	

Information about hospital				
Bed size				0.0174^∗^
Small	6260 (12.26)	4961 (12.47)	1299 (11.51)	
Medium	11869 (24.22)	9241 (24.15)	2628 (24.49)	
Large	30725 (63.10)	23944 (62.98)	6781 (63.49)	
Unknown	210 (0.42)	155 (0.40)	55 (0.51)	

Region				0.001^∗^
Northeast	9199 (19.56)	7206 (19.64)	1993 (19.30)	
Midwest	12092 (25.20)	9368 (25.02)	2724 (25.85)	
South	18311 (36.63)	14199 (36.35)	4112 (37.63)	
West	9462 (18.60)	7528 (18.98)	1934 (17.22)	

Location/teaching status				0.4155
Rural	7197 (14.66)	5623 (14.69)	1574 (14.55)	
Urban nonteaching	21877 (44.27)	17092 (44.27)	4785 (44.24)	
Urban teaching	19780 (40.65)	15431 (40.64)	4349 (40.70)	
Unknown	210 (0.42)	155 (0.40)	55 (0.51)	
Severity of illness				<0.0001^∗^

Minor and moderate loss of function	35709 (72.76)	28545 (74.51)	7164 (66.54)	
Major and extreme loss function	13355 (27.24)	9756 (25.49)	3599 (33.46)	

Manifestation of diabetes^b^				
Without any manifestation	9781 (19.94)	0	9781 (90.93)	
Renal manifestation	280 (0.57)	0	280 (2.58)	
Ophthalmic manifestation	181 (0.37)	0	181 (1.69)	
Neurological manifestation	473 (0.96)	0	473 (4.38)	
Other manifestations^c^	238 (0.48)	0	238 (2.18)	

Comorbidities				
Hypertension	28161 (57.39)	19752 (51.55)	8409 (78.16)	<0.0001^∗^
Congestive heart failure	4471 (9.13)	2995 (7.82)	1476 (13.78)	<0.0001^∗^
Obesity	3778 (7.69)	2177 (5.67)	1601 (14.85)	<0.0001^∗^
Peripheral vascular disorders	2046 (4.17)	1372 (3.57)	674 (6.29)	<0.0001^∗^

Pulmonary circulation disorders	805 (1.65)	584 (1.53)	221 (2.07)	0.0001^∗^
Renal failure	2595 (5.31)	1515 (3.97)	1080 (10.07)	<0.0001^∗^
Other cancer	1726 (3.53)	1363 (3.57)	363 (3.37)	0.3131

^a^ includes Asian, Pacific islander, native American, and others. ^c^ includes peripheral circulatory disorder, ketoacidosis, coma, and others. ^∗^ indicates statistical significance between patients with and without diabetes (*p* < 0.05).

**Table 2:** Short-term postoperative outcomes in stage I/II colon cancer patients with or without diabetes.

	Total	Diabetes		*p* value
		No	Yes	
	*n* = 49,064	*n* = 38,301	*n* = 10,763	
	(Weighted *N* = 242,136)	(Weighted *N* = 189,017)	(Weighted *N* = 53,119)	
In-hospital mortality	897 (1.82)	723 (1.88)	174 (1.62)	0.0739

Total postoperative complications^a^	13838 (28.17)	10850 (28.29)	2988 (27.72)	0.2486
Wound complications	1486 (3.02)	1169 (3.04)	317 (2.94)	0.6027
Medical complications	4547 (9.28)	3487 (9.12)	1060 (9.87)	0.0182^∗^
Surgical complications	10145 (20.64)	8034 (20.94)	2111 (19.56)	0.0019^∗^

Hospital LOS^b^				
Mean ± SE	8.21 ± 0.04	8.12 ± 0.04	8.51 ± 0.07	<0.0001^∗^
>7 days	18436 (38.30)	14001 (37.29)	4335 (41.89)	<0.0001^∗^
>10 days	9839 (20.42)	7483 (19.91)	2356 (22.24)	<0.0001^∗^
>14 days	4779 (9.94)	3663 (9.77)	1116 (10.54)	0.0237^∗^

^a^ includes wound complications, general medical complications, and general surgical complications. ^b^Hospital LOS: hospital length of stay; excludes patients who died during hospitalization. ^∗^ indicates statistical significance between patients with and without diabetes (*p* < 0.05).

**Table 3:** Identification of factors associated with in-hospital mortality in stage I/II colon cancer patients via univariate and multivariable logistic regression analyses.

	In-hospital mortality	
	Crude OR (95% CI)	Adjusted OR (95% CI)
Diabetes (yes vs. no)	0.86 (0.73-1.02)	0.70 (0.59-0.83)^∗^
Demographic features		
Age, year	1.08 (1.07-1.09)^∗^	1.05 (1.04-1.06)^∗^
Female (vs. male)	0.81 (0.71-0.92)^∗^	0.74 (0.65-0.86)^∗^

Race (vs. white)		
Black	1.05 (0.81-1.35)	
Hispanic	0.97 (0.70-1.33)	
Other ethnic groups^a^	1.05 (0.77-1.43)	
Unknown	0.91 (0.78-1.07)	

Household income (vs. Q1)		
Q2	0.97 (0.82-1.16)	0.94 (0.78-1.12)
Q3	0.87 (0.72-1.05)	0.86 (0.71-1.04)
Q4	0.75 (0.62-0.92)^∗^	0.75 (0.61-0.92)^∗^
Unknown	0.83 (0.50-1.37)	0.95 (0.57-1.60)

Information about hospital		
Bed size (vs. large)		
Small	0.89 (0.72-1.11)	
Medium	1.11 (0.95-1.30)	
Unknown	2.15 (0.96-4.85)	

Region (vs. northeast)		
Midwest	0.84 (0.68-1.04)	
South	1.14 (0.94-1.37)	
West	0.96 (0.76-1.20)	

Location/teaching status (vs. urban teaching)		
Rural	1.10 (0.90-1.34)	
Urban nonteaching	1.23 (1.06-1.43)	
Unknown	2.37 (1.05-5.36)^∗^	
Major and extreme loss function (vs. minor and moderate)	28.22 (22.31-35.69)^∗^	19.60 (15.25-25.20)^∗^

Comorbidities		
Hypertension	0.61 (0.53-0.70)	
Congestive heart failure	5.54 (4.81-6.38)^∗^	1.30 (1.12-1.50)^∗^
Obesity	0.46 (0.32-0.65)^∗^	0.50 (0.35-0.71)^∗^
Peripheral vascular disorders	2.07 (1.62-2.64)^∗^	1.08 (0.84-1.39)^∗^
Pulmonary circulation disorders	3.54 (2.63-4.77)^∗^	1.08 (0.79-1.47)^∗^
Renal failure	3.98 (3.33-4.77)^∗^	1.34 (1.11-1.62)^∗^
Other cancer	1.24 (0.90-1.72)	

^a^ includes Asian, Pacific islander, native American, and others.^∗^ indicates statistical significance (*p* < 0.05).

**Table 4:** Identification of factors for postoperative complications in stage I/II colon cancer patients via univariate and multivariable logistic regression analyses.

	Complication after surgery	
	Crude OR (95% CI)	Adjusted OR (95% CI)
Diabetes (yes vs. no)	0.97 (0.93-1.02)	0.87 (0.83-0.92)^∗^
Demographic features		
Age, year	1.01 (1.01-1.02)^∗^	1.00 (1.00-1.01)^∗^
Female (vs. male)	0.75 (0.72-0.78)^∗^	0.75 (0.71-0.78)^∗^

Race (vs. white)		
Black	1.02 (0.94-1.10)	1.04 (0.95-1.14)
Hispanic	0.85 (0.77-0.95)^∗^	0.88 (0.78-0.98)^∗^
Other ethnic groups^a^	0.88 (0.78-0.98)^∗^	0.92 (0.82-1.03)
Unknown	0.99 (0.92-1.06)	0.99 (0.91-1.07)

Household income (vs. Q1)		
Q2	0.99 (0.94-1.06)	
Q3	1.05 (0.99-1.12)	
Q4	0.98 (0.91-1.05)	
Unknown	0.97 (0.83-1.12)	

Information about hospital		
Bed size (vs. large)		
Small	0.88 (0.82-0.96)^∗^	0.91 (0.84-1.00)^∗^
Medium	0.96 (0.89-1.03)	0.95 (0.88-1.02)
Unknown	1.19 (0.81-1.74)	1.22 (0.82-1.81)
Region (vs. northeast)		
Midwest	1.26 (1.15-1.38)^∗^	1.25 (1.13-1.38)^∗^
South	1.26 (1.16-1.36)^∗^	1.26 (1.16-1.38)^∗^
West	1.35 (1.23-1.48)^∗^	1.39 (1.26-1.54)^∗^

Location/teaching status (vs. urban teaching)		
Rural	0.97 (0.89-1.06)	0.94 (0.86-1.03)
Urban nonteaching	1.10 (1.03-1.17)^∗^	1.02 (0.96-1.10)
Unknown	1.27 (0.87-1.86)	NA
Major and extreme loss function (vs. minor and moderate)	4.84 (4.62-5.07)^∗^	5.60 (5.32-5.91)^∗^

Comorbidities		
Hypertension	0.95 (0.91-0.99)^∗^	0.92 (0.88-0.96)^∗^
Congestive heart failure	1.89 (1.78-2.02)^∗^	0.68 (0.63-0.73)^∗^
Obesity	1.12 (1.04-1.21)^∗^	1.00 (0.92-1.09)
Peripheral vascular disorders	1.16 (1.05-1.28)^∗^	0.80 (0.72-0.89)^∗^
Pulmonary circulation disorders	1.92 (1.67-2.21)^∗^	0.76 (0.66-0.89)^∗^
Renal failure	1.62 (1.49-1.76)^∗^	0.78 (0.71-0.85)^∗^
Other cancer	1.11 (0.99-1.24)	

^a^ includes Asian, Pacific islander, native American, and others. ^∗^ indicates statistical significance (*p* < 0.05).

**Table 5:** Identification of factors for hospital LOS more than 7 days in stage I/II colon cancer patients via univariate and multivariable logistic regression analyses.

	Hospital LOS > 7 days	
	Crude OR (95% CI)	Adjusted OR (95% CI)
Diabetes (yes vs. no)	1.19 (1.14-1.25)^∗^	0.99 (0.94-1.04)
Demographic features		
Age, year	1.04 (1.034-1.037)^∗^	1.03 (1.02-1.03)^∗^
Female (vs. male)	0.96 (0.923-0.995)^∗^	0.94 (0.90-0.98)^∗^

Race (vs. white)		
Black	1.31 (1.22-1.42)^∗^	1.48 (1.36-1.62)^∗^
Hispanic	1.10 (0.99-1.23)	1.40 (1.23-1.60)^∗^
Other ethnic groups^a^	0.95 (0.86-1.05)	1.26 (1.12-1.41)^∗^
Unknown	1.00 (0.94-1.07)	1.10 (1.02-1.18)^∗^

Household income (vs. Q1)		
Q2	0.85 (0.81-0.90)^∗^	0.86 (0.81-0.92)^∗^
Q3	0.78 (0.74-0.83)^∗^	0.81 (0.76-0.86)^∗^
Q4	0.71 (0.67-0.76)^∗^	0.74 (0.69-0.80)^∗^
Unknown	0.88 (0.77-1.01)	1.01 (0.86-1.18)

Information about hospital		
Bed size (vs. large)		
Small	0.87 (0.81-0.94)^∗^	0.87 (0.80-0.94)^∗^
Medium	1.01 (0.95-1.07)	1.00 (0.94-1.07)
Unknown	1.01 (0.70-1.45)	0.95 (0.63-1.43)
Region (vs. northeast)		
Midwest	1.00 (0.92-1.08)	0.89 (0.81-0.98)^∗^
South	1.02 (0.95-1.09)	0.93 (0.85-1.00)
West	0.81 (0.75-0.88)^∗^	0.74 (0.68-0.82)^∗^

Location/teaching status (vs. urban teaching)		
Rural	1.08 (1.01-1.17)^∗^	1.04 (0.96-1.14)
Urban nonteaching	1.07 (1.02-1.13)^∗^	1.05 (0.99-1.12)
Unknown	1.07 (0.74-1.54)	NA
Major and extreme loss function (vs. minor and moderate)	9.11 (8.67-9.57)^∗^	8.32 (7.86-8.81)^∗^

Comorbidities		
Hypertension	1.11 (1.07-1.16)^∗^	0.90 (0.86-0.94)^∗^
Congestive heart failure	4.06 (3.79-4.36)^∗^	0.95 (0.87-1.04)
Obesity	0.95 (0.89-1.02)	
Peripheral vascular disorders	1.78 (1.62-1.94)^∗^	1.09 (0.98-1.21)
Pulmonary circulation disorders	4.11 (3.52-4.81)^∗^	1.06 (0.89-1.26)
Renal failure	2.63 (2.43-2.86)^∗^	0.88 (0.80-0.98)^∗^
Other cancer	1.44 (1.31-1.58)^∗^	1.17 (1.05-1.31)^∗^

^a^ includes Asian, Pacific islander, native American, and others. ^∗^ indicates statistical significance (*p* < 0.05).

**Table 6:** Short-term postoperative outcomes in stage I/II colon cancer patients with or without diabetes stratified by severity of illness.

	Minor and moderate loss of function		Major and extreme loss function	
	Without diabetes	With diabetes	Without diabetes	With diabetes
	*n* = 28,545	*n* = 7,164	*n* = 9,756	*n* = 3,599
	(Weighted *N* = 140,828)	(Weighted *N* = 35,345)	(Weighted *N* = 48,189)	(Weighted *N* = 17,775)
In-hospital mortality	61 (0.17)	21 (0.06)	662 (4.93)	153 (1.15)^∗^
Postoperative complications	5490 (15.34)	1267 (3.54)^∗^	5360 (40.10)	1721 (12.88)^∗^
Hospital LOS > 7 days	6996 (19.61)	2003 (5.61)^∗^	7537 (56.43)	2543 (19.02)^∗^

^∗^ indicates statistical significance compared to their counterparts without diabetes (*p* < 0.05).

**Table 7:** Short-term postoperative outcomes in stage I/II colon cancer patients with or without comorbid diabetes in the presence or absence of manifestation.

	Without diabetes	Uncomplicated diabetes	Complicated diabetes
	*n* = 38,301	*n* = 9,781	*n* = 982
	(Weighted *N* = 189,017)	(Weighted *N* = 48,301)	(Weighted *N* = 4,818)
In-hospital mortality	723 (1.88)	150 (1.53)^∗^	24 (2.49)
Postoperative complications	10850 (28.29)	2652 (27.08)^∗^	336 (34.15)^∗^
Hospital LOS > 7 days	14533 (37.97)	4017 (41.10)^∗^	529 (53.65)^∗^

^∗^ indicates statistical significance compared to patients without diabetes (*p* < 0.05). Uncomplicated diabetes: diabetes without any manifestation. Complicated diabetes: diabetes with manifestation.

**Table 8:** Short-term postoperative outcomes in stage I/II colon cancer patients with or without comorbidity.

	Without any comorbidity	With diabetes only	With diabetes and hypertension only
	*n* = 15,598	*n* = 1,465	*n* = 2,206
	(Weighted *N* = 76,972)	(Weighted *N* = 7,223)	(Weighted *N* = 10,870)
In-hospital mortality	244 (1.55)	21 (1.43)^a^	36 (1.64)
Postoperative complications	4156 (26.54)	397 (27.03)	611 (27.80)
Hospital LOS > 7 days	5092 (32.62)	546 (37.31)^a^	957 (43.31)^a,b^

^a^ indicates statistical significance compared to patients without any comorbidity (*p* < 0.05). ^b^ indicates statistical significance compared to patients with diabetes only (*p* < 0.05).

## Data Availability

The data used to support the findings of this study are included within the article.
